# A Tractable Experimental Model for Study of Human and Animal Scabies

**DOI:** 10.1371/journal.pntd.0000756

**Published:** 2010-07-20

**Authors:** Kate Mounsey, Mei-Fong Ho, Andrew Kelly, Charlene Willis, Cielo Pasay, David J. Kemp, James S. McCarthy, Katja Fischer

**Affiliations:** 1 Queensland Institute of Medical Research and Australian Centre for International and Tropical Health and Nutrition, University of Queensland, Brisbane, Queensland, Australia; 2 Menzies School of Health Research, Charles Darwin University, Darwin, Northern Territory, Australia; 3 Department of Employment, Economic Development and Innovation, Centre for Advanced Animal Science, University of Queensland, Gatton, Queensland, Australia; 4 Griffith Medical Research College, a joint program of Griffith University and the Queensland Institute of Medical Research, QIMR, Herston, Queensland, Australia; Mahidol University, Thailand

## Abstract

**Background:**

Scabies is a parasitic skin infestation caused by the burrowing mite *Sarcoptes scabiei*. It is common worldwide and spreads rapidly under crowded conditions, such as those found in socially disadvantaged communities of Indigenous populations and in developing countries. Pruritic scabies lesions facilitate opportunistic bacterial infections, particularly Group A streptococci. Streptococcal infections cause significant sequelae and the increased community streptococcal burden has led to extreme levels of acute rheumatic fever and rheumatic heart disease in Australia's Indigenous communities. In addition, emerging resistance to currently available therapeutics emphasizes the need to identify potential targets for novel chemotherapeutic and/or immunological intervention. Scabies research has been severely limited by the availability of parasites, and scabies remains a truly neglected infectious disease. We report development of a tractable model for scabies in the pig, *Sus domestica*.

**Methodology/Principal Findings:**

Over five years and involving ten independent cohorts, we have developed a protocol for continuous passage of *Sarcoptes scabiei* var. *suis*. To increase intensity and duration of infestation without generating animal welfare issues we have optimised an immunosuppression regimen utilising daily oral treatment with 0.2mg/kg dexamethasone. Only mild, controlled side effects are observed, and mange infection can be maintained indefinitely providing large mite numbers (>6000 mites/g skin) for molecular-based research on scabies. In pilot experiments we explore whether any adaptation of the mite population is reflected in genetic changes. Phylogenetic analysis was performed comparing sets of genetic data obtained from pig mites collected from naturally infected pigs with data from pig mites collected from the most recent cohort.

**Conclusions/Significance:**

A reliable pig/scabies animal model will facilitate *in vivo* studies on host immune responses to scabies including the relations to the associated bacterial pathogenesis and more detailed studies of molecular evolution and host adaption. It is a most needed tool for the further investigation of this important and widespread parasitic disease.

## Author Summary

Scabies, a neglected parasitic disease caused by the microscopic mite *Sarcoptes scabiei*, is a major driving force behind bacterial skin infections in tropical settings. Aboriginal and Torres Strait Islander peoples are nearly twenty times more likely to die from acute rheumatic fever and rheumatic heart disease than individuals from the wider Australian community. These conditions are caused by bacterial pathogens such as Group A streptococci, which have been linked to underlying scabies infestations. Community based initiatives to reduce scabies and associated disease have expanded, but have been threatened in recent years by emerging drug resistance. Critical biological questions surrounding scabies remain unanswered due to a lack of biomedical research. This has been due in part to a lack of either a suitable animal model or an *in vitro* culture system for scabies mites. The pig/mite model reported here will be a much needed resource for parasite material and will facilitate *in vivo* studies on host immune responses to scabies, including relations to associated bacterial pathogenesis, and more detailed studies of molecular evolution and host adaptation. It represents the missing tool to extrapolate emerging molecular data into an *in vivo* setting and may well allow the development of clinical interventions.

## Introduction

Scabies, or sarcoptic mange, is an infectious skin disease caused by the mite *Sarcoptes scabiei*. Human scabies is a widespread disease in developing regions of the world, and remains a significant problem amongst indigenous populations in developed countries [1]. Secondary bacterial infections of scabies lesions, most notably with Group A *Streptococcus* or *Staphylococcus aureus* [2], have been linked to serious complications, such as renal damage and rheumatic heart disease [3]. Although scabies is unusual in urban Australia, it is currently endemic in remote northern and central Australian Aboriginal communities and remains a major public health problem in these socially disadvantaged communities [4], [5]. Over 70% of two year old children in Australia's remote Aboriginal communities have been at least once infected with scabies, most of them acquiring the first infection as infants. Importantly, these numbers are reflected in the rates of observed streptococcal skin infections in over 80% of these children [6].

Parasitic mites of the genus *Sarcoptes* infest up to 40 different mammalian hosts across 17 families [7]. Commonly described hosts include dogs, pigs, foxes and wombats. Sarcoptic mange causes significant losses to primary industries, especially in pig herds, where it leads to decreased growth rates and subsequent reduced feed conversion efficiency [8]. Although effective control has been achieved in many regions by using macrocyclic lactones such as ivermectin, administered to sows 4 weeks before farrowing, sarcoptic mange remains common in piggeries, with reported prevalence from 20–86% [9]. Despite the economic and health significance of *S. scabiei* infestation in both human and animal populations, the pathogenesis and immune responses to this disease is not well understood [10].

Scabies has historically been a difficult disease to study. Scabies mites cannot be maintained or propagated away from their animal host, and it is difficult to collect mites in large quantities, as a typical infestation of human scabies involves fewer than twenty mites. Access to hyper-infested hosts has enabled the construction of cDNA libraries from human [11], [12] and fox [13] mite populations. Sequencing these libraries has resulted in the identification of genes which reveal some unexpected features of scabies biology and immunopathology [reviewed in 14]. We have recently shown that scabies mite gut proteases play major roles in maintaining the mite infestation within the epidermis, either as digestive enzymes [15], or by providing host complement evasion mechanisms [16]. Another research focus has been the characterisation of the molecular basis of emerging acaricide resistance [17], [18].

Despite these recent advances, access to hyper-infested hosts remains opportunistic and sporadic. Moreover, there has been an almost complete failure in efforts to maintain viable mites in the laboratory for longer than 24–48 hours, and no established methods are available to propagate mites *in vitro*. To overcome these barriers, the availability of a tractable animal model for scabies would be of enormous benefit. Despite being morphologically very similar [19], *S. scabiei* variants appear to be predominantly host specific, and investigations whether they are genetically distinct are ongoing [20], [21]. The majority of cross-infectivity studies have been unsuccessful, with infestations transient and self limiting [22]. Over several years we have tried unsuccessfully to propogate mites from crusted scabies patients, pigs and dogs on immunosuppressed mice. There is currently only one other animal model for scabies worldwide, that of canine scabies mites maintained on rabbit hosts at a U.S laboratory [22]. This trans-species adaptation was successful only after many attempts, and has not been able to be repeated (personal communication, L. Arlian Ohio). While this host-mite system has been extremely valuable for immunological studies, it has been maintained under strong drug selection pressure for over two decades [18], and consequently its utility as a source of live, viable mites is limited due to logistical difficulties of access for international research and regulatory restrictions.

When infested with mange, pigs show similar epidermal, morphological, and immunological changes to humans [23]. Of significant importance is the fact that pigs have been described to develop a manifestation of scabies closely resembling human crusted scabies [24], a poorly understood manifestation. A further potential advantage of this host-parasite system is the fact that the complement system in pigs is comparable to humans [25], and pigs are susceptible to Streptococci, including Group A streptococci (GAS) [26]. Experimental models of porcine mange have been used previously to study clinical manifestations [27], effects on production and transmission dynamics, as well as limited studies of immunology and histopathology [28]. The majority have been short-term, naturally resolving infections. Our objective was to achieve a consistent, reliable quantity of *S. scabiei*. Herein we report progress in developing a sustainable experimental model of chronic porcine mange, which now consistently provides large numbers of mites, facilitating the less restrictive conduct of research on human and animal scabies.

## Methods

### Ethics Statement

All animals were handled in strict accordance with good animal practice as defined by the Australian code of practice for the care and use of animals for scientific purposes and the NHMRC's Animal Code of Practice, and all animal work conducted with ethical approval from both DEEDI and QIMR Animal Ethics Committees (DEEDI-AEC SA2009/07/294, QIMR A0306-621M).

### Mite collections from abattoir samples

Samples were initially sought from pigs for the purpose of obtaining sufficient mites to attempt transmission of infection to mice. Six ears from slaughtered pigs with mangy appearance were supplied to the laboratory in a weekly basis by a Southeast Queensland abattoir from June 2004 to November 2007. Skin pieces were dissected from the inner ear and incubated in glass petri dishes at 27°C, which encourages mites to crawl out towards the heat source. Dishes were examined and mites picked under a dissecting microscope using microscopic needles.

### Pig Housing

Pigs were housed at the DEEDI Animal Research Institute, Yeerongpilly, QLD, and at the Centre for Advanced Animal Studies, Gatton, QLD. It was ensured that the care and the experimental practices conformed to the Australian animal ethics guidelines. Pigs with suspected natural mange infections (Table 1, groups 1–4) were sourced by approaching commercial piggeries, saleyards and private owners. At a later stage of the study (groups 5–10) naïve piglets of the “large white” breed - a common meat producing breed in Australia [29] - were obtained from the University of Queensland piggery, Gatton, QLD and experimentally infected with mites. To passage the infection, new piglets were placed into pens adjacent to infected pigs from the previous group. A heater placed on the fence line encouraged pigs to congregate and thus enhanced the potential for mite transfer. From groups 6 onwards, mite transmission was additionally ‘boosted’ by the direct transplant of mite infested skin crusts obtained directly from the previous group. Briefly, crusts were harvested from infected pigs and dissected into small pieces (approx 0.5cm^2^). Several crusts were inserted into both ear canals of naïve piglets. Pigs were temporarily restrained to prevent dislodgement of the crust by agitation, thereby allowing successful infestation.

**Table 1:** Development of an animal model for scabies, 2004–2009.

Year	Group # (number of pigs)	Source of pigs	Arrival age (weeks)	Source of infection	DEX treatment	Mites per harvest (max)^3^	Duration of infection (weeks)
2004	1 (1)	Boonah, QLD	60	Natural	None	0	0
2004	2 (5)	Oakey, QLD	12	Natural	None	19	8
2004	3 (4)	Cooyar, QLD	4	Natural	0.01mg/kg, injection, 1/wk	50	10
2006	4 (4)	Boney Mountain, QLD	3	Natural	0.01mg/kg, injection,3/wk	150	12
2006	5 (3)	Piggery, UQ Gatton	3	Passaged^1^	Pre-treated, 0.1mg/kg,injection, 3/wk	300	12
2007	6 (4)	Piggery, UQG	3	Passaged+Boosted^2^	Pre-treated, 0.1mg/kg, oral, 3/wk-daily	500	40*
2007	7 (3)	Piggery, UQG	3	Passaged+Boosted	Pre-treated, 0.1mg/kg, oral, daily	>1000	52*
2008	8 (3)	Piggery, UQG	3	Passaged+Boosted	Pre-treated, 0.25–0.3mg/kg, oral, daily	>10,000	20*
2009	9 (2)	Piggery, UQG	3	Boosted	Pre-treated, 0.2mg/kg, oral, daily	100,000	44*
2009	10 (3)	Piggery, UQG	3	Passaged+Boosted	Pre-treated, 0.2mg/kg, oral, daily	100,000	14*

1 Passaged: infection transmitted by housing mange infected pig with naïve pig.

2 Boosted: infection transmitted by transplant of mite infested skin crusts.

3 Average harvest involved scraping 4cm^2^ ear section.

*Pigs still infected at time of euthanasia.

### Sampling of mites

Skin samples were collected by gently scraping and lifting off encrusted areas from the inner ear area of the pig with a sharpened teaspoon and subsequently examined for mites.

### DNA preparation, sequencing and data analysis

*S. scabiei* var. *suis* DNA from 10 mites collected from one pig in group 4 in 2006 and from another pig in a later group 10 in 2010 was prepared as described previously [30]. The SMIPP-S-B2 gene was amplified in a proofreading PCR and products were purified and sequenced in both directions, with chromatograms manually inspected for quality. These sequences were then aligned with SMIPP-S-B2 (AY333073), with the related SMIPP-S-B1 (AY333076), and SMIPP-S-B3 from *S. scabiei* var. *hominis* cDNA used as outgroups (data not shown). Sequence alignments and subsequent phylogenetic analysis was performed using MEGA4 [31].

## Results and Discussion

### Mite supply from abattoir collections is sporadic

**Figure 1:**
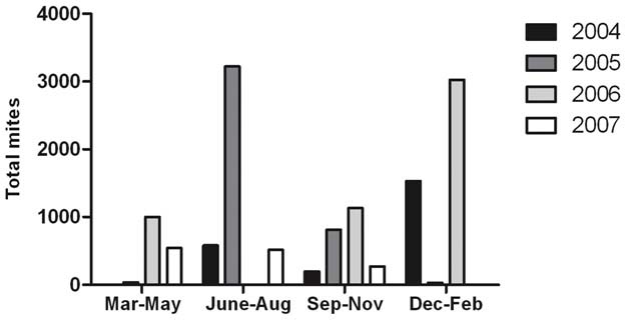
Mites collected from south-east Queensland abattoir, 2004–2007. Mites were obtained weekly from six pig ears and total numbers of mites were counted. The graph shows the trend of mite numbers for each year and season (June–August = Australian winter months).

The quantity of mites obtained from abattoir samples was variable, exceeding 3,000 mites per ear on some occasions, whereas at other times no mites were observed for several months (Figure 1). Although seasonal trends for mange occurrence have been reported [32], this trend is likely dependent on other determinants including housing conditions, with pigs in confinement showing less seasonal differences in mite burden [33]. While there is no obvious seasonal trend apparent from our data (Figure 1), without having prior knowledge of the source environment from the samples obtained, we could not investigate for any association. The results however accord with the known prevalence of sarcoptic mange in the pig industry, despite the availability of effective control measures. The sporadic supply of mites obtained from this approach meant that it was unsuitable as a sustainable source for laboratory experiments. Clearly, an alternative approach was needed.

### Dexamethasone treatment increases mite burden in pigs

Despite having clinical features of mange, such as scratching behaviour, hair loss, reddened and flaky skin, the untreated pigs in groups 1 and 2 either had no or very few mites and symptoms resolved within 6 weeks of acquisition (Table 1). These observations are typical for acute-hypersensitive mange in pigs, which is generally described as a short term infestation with low mite numbers [27], a clinical picture akin to ordinary human scabies. To obtain sufficient mites for our studies, pigs with chronic mange were needed. Chronic mange closely resembles human crusted scabies, with the formation of hyperkeratotic plaques and proliferation of mites. It is more commonly observed in older sows or when pigs are immunosuppressed, and is generally uncommon in growing pigs [27], [33].

To increase mite numbers and maintain a prolonged infestation by counteracting natural immunity, it was proposed to treat mange infected pigs with corticosteroids. This concept is supported by the observation that corticosteroid therapy often results in the development of crusted scabies in humans [34]. Dexamethasone is a synthetic glucocorticoid commonly used in animal models to promote infection and exacerbate infectious responses. It has pleiotropic effects on immune responses, depressing lymphocyte production, antibody production and inflammatory responses. Due to its previous use in other immunosuppression models, including pigs [35], [36], it was selected for study.

Because dexamethasone had not been tested previously in scabies infected pigs, and to give appropriate consideration to animal welfare issues, a gradual, conservative treatment program was initiated.

As naturally infected pigs self cured over time (Table 1, Groups 1 and 2) pigs with existing, naturally acquired mange were treated (Table 1, Group 3) with weekly injections of 0.01mg/kg of dexamethasone (Dexafort, Provet, Brisbane). However this did not increase mite numbers or prolong infection. Mite numbers improved markedly when dexamethasone was given tri-weekly but the infestation was not sustained for longer than 12 weeks and only a single viable mite harvest was possible from each pig (Table 1, group 4). Because of the possibility that the immune system was overcoming infection prior to steroid treatment, dexamethasone treatment was commenced in naïve piglets prior to infection. In light of marginal effects observed at lower dosage, the dexamethasone dosage and frequency was increased to 0.1mg/kg daily. For these groups (5 and 6) a successful experimental transfer was observed, with up to 500 mites obtained in a single ear scraping. Because of concerns regarding aversion behaviours to daily injections, the delivery method of dexamethasone delivery was changed from injection (groups 3–5) to oral (Dexamethasone tablets, 1 or 4mg, Provet, Brisbane), with pigs readily accepting tablets offered in marshmallows. A further alteration (applied to groups 6–10) was to boost the passage of mites by directly placing mite infested skin crusts deep into the ear of piglets. Combined, these protocol modifications greatly enhanced rate of infection, with new piglets developing crusted lesions within four weeks. Mite numbers continuously increased, and one pig maintained infection for over six months (Table 1, Group 7).

**Figure 2:**
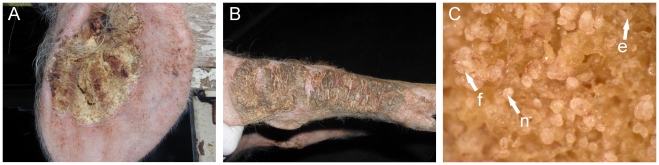
Chronic mange in immunosuppressed pigs. (A) Typical encrustment at peak of infection on pig ear (B) and spread of encrusted lesions to hocks as a result of increased dexamethasone dosage in group 8. In excess of 6000 mites per gram of skin are obtained. (C) A close up on piece of crust obtained from a typical harvest viewed through dissecting microscope showing large numbers of females (f), nymphs(n), and eggs (e).

Since 0.1mg/kg appeared to be inadequate to achieve sufficient immunosuppression, moderate increments in dosage were commenced, whereby a sentinal pig was exposed to the higher dose for six weeks prior to the rest of the group, allowing for observation of detrimental effects. At 0.3mg/kg pigs started to develop much larger crusted lesions which also spread to regions beyond the ears (Figure 2B). At this point, an excess of 10,000 mites were obtained per harvest, and crusting would re-develop after removal, enabling multiple harvests (Table 1, Group 8). Given that our objective of developing sustainable mite supply was achieved, further dose increases were not undertaken.

At the maximal dose of 0.3mg/kg dexamethasone mite infestation dramatically increased, but noticeable side effects were observed. The most obvious of these was a tendency of growth retardation and a change of body shape. Retardation in weight gain in dexamethasone treated pigs has been reported [36]. Decreased growth can be attributed to the interference of the corticosteroid on the hypothalamic-pituitary axis, thus depressing production of growth hormone. Stunting was not associated with decreased nutrition uptake, and all pigs had healthy appetites. At higher doses some pigs also developed hirsutism. Several pigs developed laxity in hind foot tendons, and in one pig evidence of excess adiposity and bone demineralisation was observed on post mortem. Frequent vomiting was observed in one pig. While dexamethasone is known to increase the risk of gastric ulcers, no evidence of ulcers or gastrointestinal irritation were observed post-mortem. Corticosteroids have been used to induce stress responses, and this was observed in several pigs having decreased ability to cope with bullying and natural dominance behaviours. This effect was controlled by housing pigs in pairs or singly, thereby minimising the requirement to compete for social positioning.

Most these side effects parallel that of iatrogenic Cushing's syndrome seen in humans, with glucocorticoid excess resulting in symptoms such as centralised adiposity, bone osteoporosis, hirsutism, depression and anxiety. It should be emphasised that the side effects observed in this study were mild, and aside from the physical effects, pigs appeared normal in spite of their mange infestation. Pigs were closely monitored by skilled veterinary staff for side-effects including increased susceptibility to disease, but other than mange, no additional infections were observed. Good husbandry and infection control measures were paramount to this process.

Since the optimal treatment regimen has now been established, the dosage of dexamethasone can be adjusted accordingly to maintain required mite burden, while minimising side effects. Continued tailoring of dexamethasone is also important since it became evident through the trial that pigs showed great variation in inherent immunologic responses to both steroid treatment and scabies. For example, one pig on low dose dexamethasone still developed chronic mange and the side effects described above. Similarly, other pigs had relatively low mite burdens despite receiving higher doses of dexamethasone. Variation in pig mange responses are common [27], with small number of pigs in the population often harbouring the majority of the mite burden [8].

Our data (summarized in Table 1) suggest the development of a stable and transmissible mite population established from an originally natural infection over five passages. We observed a striking increase in mite numbers per harvest especially for the last three groups.

### Comparative phylogenetic analysis of mites collected from naturally infected pigs versus pig mites collected a recent cohort of the pig/mite model

We propose that the increased mite numbers are at least in part a direct consequence of the immunosuppression regimen and reflect the immune status of the pigs. It is however also possible that the selection towards adapted subpopulations of mites has occurred via passage through five generations of pigs. Any adaptation of the mite population should be reflected in genetic changes. Such changes over time can only be observed in a mite population within a closed cohort, where there had been no introduction of mites with potentially new genotypes. As a indispensable prerequisite for such a study the presented pig/mite model provides a continuous source of mites and may offer a controlled setting to monitor genetic changes over time in closed mite populations. To test the feasability of the model for a large experimental setup at a later stage we aimed here to compare limited sets of genetic data obtained from pig mites collected from a naturally infected pig in group 4 (2006) with data from pig mites collected from one pig in group 10 (2009). Thus, we were investigating genetic changes in a closed mite population maintained isolated for over 3 years.

We focussed on the SMIPP-S-B2 gene, belonging to a multigene family of at least 32 closely related gut proteases homologous to the group 3 major allergens of astigmatid house dust mites [37] [38]. Due to mutations in the catalytic triad and considerable structural rearrangements, these have no proteolytic function, but have instead evolved into potent inhibitors of each of the three pathways of the host complement system [16]. The genes in the SMIPP-S family show considerable sequence diversity, and are hypothesised to mediate a novel host immune-evasion strategy. Synonymous versus non-synonymous changes within the coding region of the mature SMIPP-S protein indicate that SMIPP-Ss most likely have functions that would be affected by non-synonymous changes and therefore be subjected to selection pressure [37].

**Figure 3:**
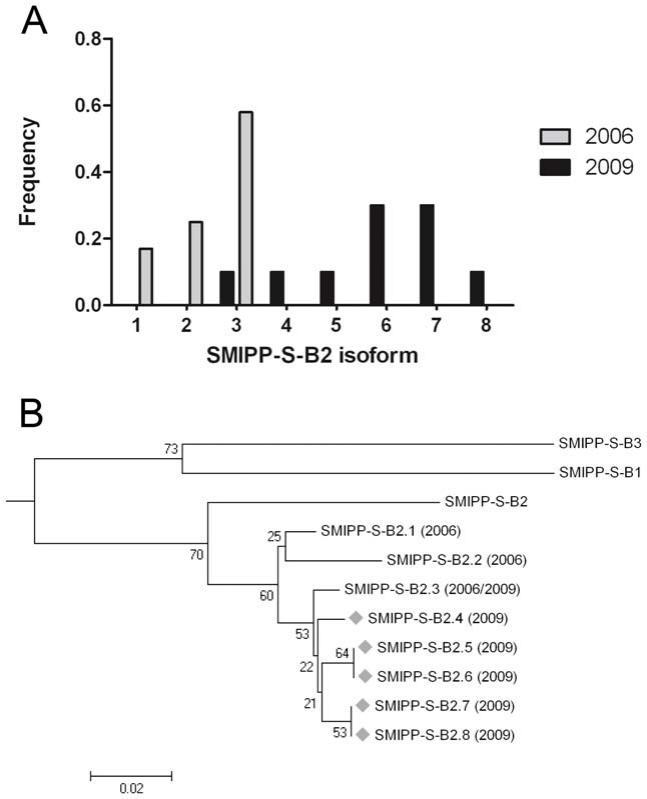
Genetic changes within a subset of the SMIPP-S multigene family in a closed mite population. A: Frequency histogram of the SMIPP-S-B2 sequence isoforms identified in pig mites in 2006 (grey) and 2009 (black). B: Neighbour-joining bootstrap tree (500 replicates) showing the type-B subfamily of the scabies mite inactivated serine protease paralogues (SMIPP-Ss). A multiple sequence alignment was performed with ClustalW, using the sequences obtained from the *S. scabiei* var. *hominis* cDNA (SMIPP-S-B1, SMIPP-S-B2 and SMIPP-S-B3) and the SMIPP-S-B2 pig mite sequences identified in 2006 and 2009 (SMIPP-S B2.1–2.8). New isoforms identified in 2009 are designated by the diamond symbol. Whole numbers at nodes indicate the percentage of bootstrap support. The tree is drawn to scale, with branch lengths corresponding to evolutionary distances. Phylogenetic analysis was conducted using MEGA4 [31].

The pig mite derived SMIPP-S-B2 sequences form a distinct cluster from the corresponding human mite sequence. We also observed a considerable degree of intra-species heterogeneity. In the 2006 mite population, three distinct B2 isoforms (B2-1 to 3) were observed, with polymorphisms at 7 amino acid sites (Figure 3A) with isoform B2-3 observed most frequently. Analysis of the 2009 mite population revealed the emergence of five new isoforms (B2-4 to 8), with isoforms B2-1 and 2 not seen in the 2009 mites. Neighbour-joining analysis suggests these new isoforms are derived from B2-3 (Figure 3B). Some of the bootstrap values were low, which is most likely attributable to the proposed recent evolutionary divergence, also indicated by the short branch lengths at the respective nodes. Indeed, the primary source of sequence variation involved polymorphisms at amino acid residue 184. Whereas human mite sequences contain an alanine at this site, pig mites in 2006 contained threonine or aspartic acid, and 2009 pig mites had alanine, threonine, aspartic acid or glutamate. Sequence alignments of the complete SMIPP-S family show that this region is highly polymorphic [38]. Moreover, the predicted surface location of this residue suggests this rapidly evolving divergence may be of functional relevance. It is suggested the SMIPPs provide a complex network of immunologically cross-reactive sequences in response to host antibodies, and thus represent specific genetic adaptations to the intimate host-parasite relationship [37].

While heterogeneity within the SMIPP family most likely happens *in vivo* in human and pig populations, it is difficult to undertake such studies in human settings. In previous genetic analysis on mites from human crusted scabies, patients had been infected over many years and had undergone multiple treatments over several episodes. In microsatellite data reported by Walton *et al.* [20], [39] a large degree of genetic diversity in the mite population within individual hosts was possibly the result of both recrudescence and re-infection from multiple community sources. In contrast our animal model provides a controlled setting to monitor genetic changes over time in closed mite populations.

Although this SMIPP-S analysis was limited in its scope, it nevertheless highlights the potential of this model for investigations of the molecular evolution of scabies mite genes. Representative isolates collected over the continuing passage of mites through successive generations may provide fascinating insights into genetic diversity, evolution and host-adaption of scabies mites, particularly within the novel SMIPP family.

In conclusion, over a prolonged timeframe of five years, involving ten independent cohorts (32 animals in total), we have successfully developed a sustainable experimental infestation of scabies mite on immunosuppressed pigs. Encrustment on the ears occurs after 6–12 weeks and can be maintained for at least 12 months, depending on drug dosage and individual pig response. This animal model now consistently provides large mite numbers (>6000 mites/g skin) for molecular-based research on scabies. Projects that have benefitted from this to date include detailed studies of gene expression in scabies mites [40], and the development of new techniques to measure drug sensitivity (submitted), pilot studies in preparation to sequence the scabies mite genome, and histological localisation of various molecules involved in evasion of host defences [15], [16], [41].

Most importantly, further research may utilise the full features of this animal model which facilitates *in vivo* studies, including investigation of pig immune responses to scabies and dexamethasone pathways of immunosuppression, in addition to more detailed studies of molecular evolution and host adaption. Furthermore, our pig model is now at a stage to be optimised as pig/scabies/GAS model. Such research should result in innovative tools for the prevention, monitoring and further investigation of this important and widespread parasitic disease.
